# The Impact of Activity Based Working (ABW) on Workplace Activity, Eating Behaviours, Productivity, and Satisfaction

**DOI:** 10.3390/ijerph15051005

**Published:** 2018-05-17

**Authors:** Lauren Arundell, Bronwyn Sudholz, Megan Teychenne, Jo Salmon, Brooke Hayward, Genevieve N. Healy, Anna Timperio

**Affiliations:** 1 Institute for Physical Activity and Nutrition (IPAN), School of Exercise and Nutrition Sciences, Deakin University, Geelong, VIC 3220, Australia; b.sudholz@deakin.edu.au (B.S.); megan.teychenne@deakin.edu.au (M.T.); jo.salmon@deakin.edu.au (J.S.); anna.timperio@deakin.edu.au (A.T.); 2 Ko Awatea, Counties Manukau Health, Auckland 2025, New Zealand; Brooke.Hayward@middlemore.co.nz; 3 School of Public Health, The University of Queensland, Brisbane, QLD 4072, Australia; g.healy@sph.uq.edu.au; 4 Baker IDI Heart and Diabetes Institute, Melbourne, VIC 3004, Australia; 5 School of Physiotherapy, Faculty of Health Sciences, Curtin University, Bentley, WA 6102, Australia

**Keywords:** workplace, work environment, sedentary behaviour, sitting, eating behaviours, productivity, activity-based working

## Abstract

The redesign of the physical workplace according to activity-based working (ABW) principles has potential to influence employee health and workplace outcomes. This natural experiment examined changes in accelerometer-derived workplace activity, self-reported eating behaviours, productivity, workplace satisfaction before (March to November 2014) and six to nine months after moving to an ABW workplace compared to a comparison workplace (*n* = 146 at baseline (56% ABW, aged 40.1 ± 8.5 years, 72% female). Interviews were also conducted with 21 ABW participants. Between- and within-group differences were examined and mixed model analysis examined intervention effects over time. Effect sizes were calculated on change scores (Cohen’s *d*). Although not statistically significant, ABW participants had meaningful improvements in workday sedentary time, light-, and moderate-to-vigorous intensity physical activity, job satisfaction and relationship with co-workers (*d* = 0.379–0.577), and small declines in productivity (*d* = 0.278). There were significant, meaningful, and beneficial intervention effects on perceived organisational support for being active in the workplace, frequency of eating lunch with colleagues, and satisfaction with the physical environment in ABW compared to comparison participants (*d* = 0.501–0.839). Qualitative data suggested that ABW employees associated ABW with greater opportunities for movement and collaboration, but had mixed views on the impact on productivity. Future research with larger samples and over longer follow-up periods is warranted.

## 1. Introduction

Inadequate physical activity and high amounts of sitting contribute to an array of chronic health conditions, such as cardiovascular disease, diabetes, cancer, and associated participation in detrimental health behaviours, such as poor diet [1,2]. A key setting in which to address these behaviours is the workplace [3]. Recent findings indicate that, on average, office-based workers spend 75% of their workday sitting [4,5], with much of this time accrued in prolonged, unbroken bouts [6] which may be particularly detrimental to health [7,8]. A key influence on sitting and activity levels in the workplace is the physical work environment [9]. Interventions examining individual-level approaches, such as installation of activity-permissive workstations that allow employees to complete their typically seated work while standing (e.g., height-adjustable desks) or moving (e.g., treadmill desks), have shown promise in reducing workplace sitting, particularly when deployed as part of a multi-level approach to change [6,10,11,12,13] without detrimentally influencing workplace productivity [14,15].

At a broader level, redesign of the office working environment—for example to the Activity Based Working (ABW) design concept—has been gaining popularity [16]. The key design feature of ABW is the departure from traditional allocated seating to open plan offices with a variety of shared work spaces designed for different work tasks (e.g., individual and collaborative work, brainstorming, phone conversations, and meetings) [16]. Work spaces are chosen and used by employees based on their current work tasks with regular transitions between them encouraged [16]. In addition, ABW incorporates centralised facilities such as stairs and common eating areas (with associated policies prohibiting eating at the desk), new technologies (e.g., removal of corded phones and use of instant messenger), mobile devices such as laptops, and may also include height-adjustable desks [16]. Although originally designed to enhance collaborative working while reducing office space requirements and operating costs [16], ABW has the potential to favorably influence a variety of health [8], behavioural, and work-related outcomes [17]. However, to date, there has been minimal evaluation of the impact of ABW on workplace activity (including organizational support for being physically active in the workplace), eating behaviours, and productivity and workplace satisfaction.

Two studies have examined the impact of building design on changes in employees’ accelerometer- and inclinometer-measured physical activity and sedentary behaviour, examining these behaviours before and after relocating to a building specifically designed to enhance activity using active design principals [18,19]. Here, the building was specifically designed to encourage more movement, for example, through centralization of stairwells and vertical integration [18,19]. However, ABW principals were not necessarily applied throughout. Reductions in time spent sitting (by approximately 20 min) [18,19], standing (by approximately 20 min) [18,19], and light- intensity physical activity [18] were observed after four months, each with moderate effect sizes. However, both studies included small sample sizes (*n* = 24 [19] and *n* = 42 [18]) and neither had a control group for comparison. Two studies have specifically examined examine changes in physical activity and sedentary behaviours within an ABW designed workplace. One study (*n* = 88) found small improvements in accelerometer measured sedentary time, light- and moderate-intensity physical activity during a four-week trial move to an ABW environment [20]. However, behaviours returned to baseline levels once employees moved back to their original (traditional) office [20]. Interestingly, after returning to their traditional desks, employees self-reported reductions in sitting and increases in their standing and walking. Over the four-week trial, there was no change in employees’ self-reported work ability [20], yet when these employees returned to their traditional office, self-reported work ability declined suggesting a preference for the ABW workplace. The second study included a control group (*n* = 79 intervention and *n* = 31 control participants) but found no significant changes to workplace sitting time at 3- or 12-months post relocation to an ABW designed office [21]. While the literature examining the impact of ABW design on workplace movement is still in its infancy, the findings provide preliminary indications that the physical environment is important for workplace movement and productivity. In addition, ABW-oriented workplace design and policy may affect employees’ eating behaviours, organisational support for being physically active in the workplace, and workplace satisfaction; however, this is yet to be examined.

Studies examining the impact of ABW designed workplaces on a range of outcomes is rare, and research designs are limited by short follow-up periods and a lack of a comparison group. The aim of this study was to explore the impact of ABW on employees’ workplace activity, eating behaviours, productivity, and workplace satisfaction. The study also aimed to qualitatively explore employees’ perceptions of the impact of ABW on these outcomes.

## 2. Materials and Methods 

### 2.1. Study Design and Procedure 

This natural opportunistic experiment, conducted in one local government area (LGA) in Victoria, Australia, involved a quasi-experimental design (pre-post design with a comparison group). The ABW principles were implemented in a new purpose-designed building in one municipal workplace. A nearby convenience comparison municipal workplace from the same LGA was identified which offered a similar traditional office-based workplace design had no plans to change their physical workplace environment during the study period and within a similar external environment (e.g., similar urban design and walkability). The Chief Executive Officer (CEO) from each workplace provided written consent to participate in the evaluative component of the study. Information about the study was distributed via an email from the CEO and staff were reminded about the study in team meetings. Written consent was obtained from all participants. Baseline (pre-test) measures were taken in both workplaces (March to November 2014) prior to staff in the intervention group relocating to the ABW designed building. Follow-up (post-test) measures were taken six to nine months after the new ABW building was operational and all staff at the ABW workplace had moved in (May to August 2015). Assessments included completing an online questionnaire (hosted by Qualtrics), wearing an accelerometer for five workdays during waking hours, and completing a logbook to record work hours and any times when the monitor was not worn during work hours. A sub-sample of ABW participants were stratification by age and gender and then selected at random from these age/sex groups, to complete a 15–20 min face-to-face or telephone interview. Ethical approval was received from the Deakin University Human Ethics Advisory Group (Health); Project Number HEAG-H 174-2013.

### 2.2. The ABW Workplace 

Eighteen months prior to relocation to the new ABW designed building, some ABW principles [16] were gradually introduced by the Human Resources team. Firstly, employees were introduced to new mobile technologies (e.g., laptops, telephone systems) and etiquette for the working areas (e.g., ensuring quiet within a ‘Focus’ area) for the various ABW workspaces. Secondly, a number of progressive policy changes were introduced in the existing traditional building (e.g., paper-reduction strategies, restrictions on eating at desks, and encouragement to use different desks from day-to-day). 

The new ABW building incorporated an open-plan design, centralised facilities (e.g., staircases, eating hubs), new technologies and mobile devices, non-delegated seating, 12 height-adjustable desks, a paperless policy, a policy banning eating at desks and encouraging utilization of shared kitchens, and different workspaces that staff could choose to work at depending on the demands of their work tasks. These workspaces were designed for eight types of work: focus (individual tasks requiring concentration and silence), process (individual tasks involving interactions), chat (informal sharing of information, two-to-four people), duo work (collaborations and sharing of computer tools, two people), brainstorming (sharing and generating ideas, small groups), video calls (conferencing and individual calls), co-ordination (formal and goal driven by a leader, ≥3 people), and information presentation (formal training, presentations, large groups). 

### 2.3. Sample Size and Participants 

**Figure 1:**
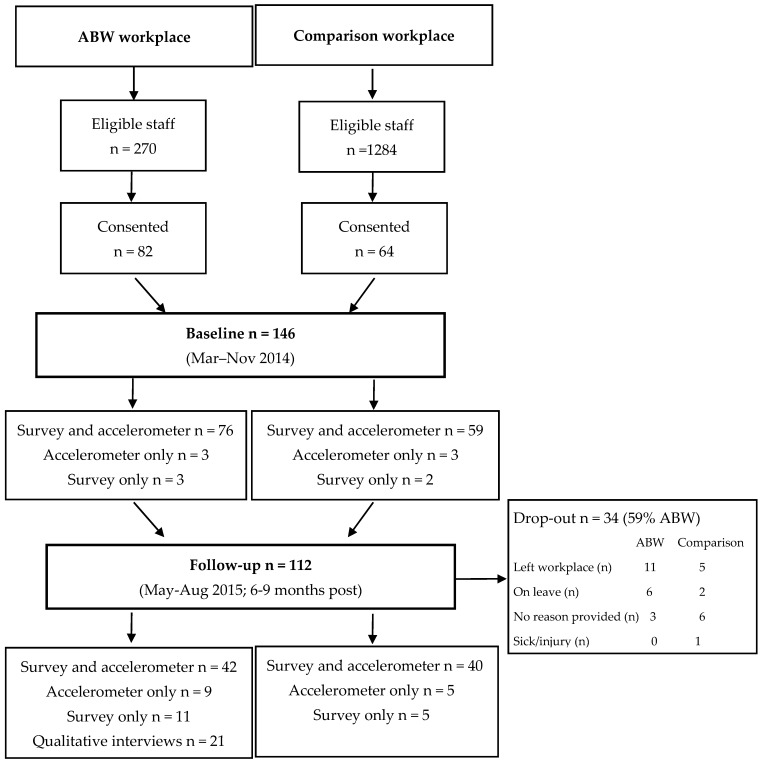
Participant flow and numbers through the study.

This was an opportunistic, exploratory study where all eligible staff at both workplaces were invited to participate (number of eligible staff at the ABW workplace *n* = 270 and at the comparison workplace *n* = 1284). Eligibility criteria included being over 18 years of age, employed primarily in an office-based job (i.e., outreach workers were ineligible), on a full- or part-time contract, and anticipation of employment at the workplace until at least March 2015. Figure 1 displays the flow of participants throughout the study. Based on response rates from previous studies examining activity permissive workplaces (e.g., 20% [20], 34% [19]), it was anticipated that 100 staff from each workplace would participate. The sample size for the interviews was obtained to ensure diversity of experiences and to ensure data saturation.

### 2.4. Objective Measure of Workplace Movement: Workplace Sedentary Time and Physical Activity

The GT3X Actigraph accelerometer (Pensacola, FL, USA) was used to measure time spent sedentary (<100 counts per minute (cpm) on the vertical axis, SED [22]), in light- (LPA; 101–2019 cpm), and in moderate-to-vigorous intensity (MVPA; 2020–9627 cpm) physical activity [23]. The accelerometers were worn on an elastic waist belt for five working days. They were removed for sleeping and water activities (e.g., shower, swimming). The accelerometer data were downloaded using Actilife Lifestyle Monitoring System Version v6.10.2 and processed using a customised Microsoft excel macro. Data were extracted for work hours only, as reported in the logbook. A number of methods were applied to impute work hours for days with incomplete logbooks at baseline and/or follow-up (*n* = 106). For part-time employees, missing work start and finish times were considered to indicate that it was a non-work day. For full-time employees, if there was evidence of consistent work times at either baseline or follow-up, these times were imputed for days on which start/end times were missing days. If there was no evidence of a consistent pattern, default work hours of 9 a.m. to 5 p.m. were used (reflective from the work times obtained). Data was imputed for 1–10 workdays across the two assessment periods: (1 day *n* = 1, 2 days *n* = 13, 3 days *n =* 0, 4 days *n* = 12, 5 days *n* = 28, 6 days *n* = 9, 7 days *n* = 0, 8 days *n* = 1, 10 days *n* = 41).

Non-wear time was defined as 60 min of consecutive zero accelerometer counts [20,23,24]. Wear time was standardised to an eight-hour workday (i.e., standardised wear time = wear time/480 min), and each behavioural outcome was standardised to this workday (e.g., sitting minutes/standardised wear time) to account for time spent working [19]. Therefore, outcome variables are expressed in minutes per eight-hour workday. To be included in analysis participants were required to have worn the monitor for at least 75% of their working hours on at least one work day at either assessment [4].

### 2.5. Survey Measures

#### 2.5.1. Socio-Demographic and Work Characteristics

Sex, age, education, work capacity, highest level of education, height, and weight were self-reported at baseline. Height and weight was used to calculate Body Mass Index (BMI: kg/m^2^), with participants then classified as below or healthy weight range < 18.5–24.99 or overweight/obese ≥ 25.0 [25].

#### 2.5.2. Organisational Support for Being Physical Active in the Workplace

Using a five-point Likert scale (‘strongly disagree’ to ‘strongly agree’), participants were asked to rate their levels of agreement with eight items pertaining to support for being physically active in the workplace over the past month, including: “My workplace is committed to supporting staff health and well-being, and staff choices to stand or move more at work”; “My colleagues/supervisors would not mind if I chose to stand up while working at my desk, stand up during a meeting, or walk over and talk to them instead of sending them an email”. Items were summed to create an overall variable for organisational support for being physically active in the workplace (range 5–40). The items have previously demonstrated adequate reliability properties [26], and showed acceptable internal reliability properties in the current study (baseline Cronbach’s α = 0.85, follow-up Cronbach’s α = 0.88).

#### 2.5.3. Workplace Eating Behaviours 

Participants also completed newly developed survey items to collect data on the number of work days in a usual week they: (1) stopped for lunch; (2) ate lunch with colleagues; and (3) snacked between meals during a usual working week. 

#### 2.5.4. Workplace Productivity 

Participants were asked to rate their performance on nine work parameters (‘amount of work accomplished’, ‘quality of work accomplished’, ‘meeting deadlines’, ‘frequency of errors’, ‘taking responsibility’, ‘creativity’, ‘getting along with others’, ‘dependability’, and ‘overall work performance’) using a 10-point scale (1 = absolutely unacceptable to 10 = absolutely ideal). Self-rated productivity was computed as the average of the nine items among those who answered ≥7 items (range 9–90). The scale was adapted from a previously developed scale with known adequate reliability and validity [27]. The scale had acceptable internal reliability properties in the current sample (baseline Cronbach’s α = 0.87, follow-up Cronbach’s α = 0.83).

#### 2.5.5. Workplace Satisfaction 

Participants were asked to rate their level of workplace satisfaction over the past week on four statements: ‘the physical workplace environment in which you work (e.g., amount of noise, temperature)’, ‘overall job’, ‘relationship with co-workers’, and ‘relationship with supervisors’ using a 10-point Likert Scale (1 ‘Very dissatisfied’ to 10 ‘Very satisfied’). These four items from the Health and Work Questionnaire have demonstrated validity for measuring the impact of interventions on workplace outcomes [28]. 

#### 2.5.6. Process Evaluation: Perceived Changes to Workplace Activity and Height-Adjustable Desk Use

ABW participants were asked additional process evaluation questions examining perceived changes to their workplace movement and their use of height-adjustable desks. At follow-up, participants were asked if the amount of time they spend at work sitting, standing, and walking/moving around, and the number of breaks or interruptions to sitting they perform at work had decreased, not changed, or increased compared to one year ago (i.e., prior to moving to the ABW workplace). ABW participants were also asked to report how frequently they used the height-adjustable desks (never, <1 day/week, 1–2 days/week, 3–4 days/week, everyday). If they used the desks, they were then asked how long they stood at them (response options: stand for most of the time; stand for some/half the time; stand for about a quarter of the time; regularly break-up sitting when using the desk, e.g., stand after every 30 min). Participants were also asked if they would like to use the height-adjustable desks more often (yes/no). 

### 2.6. Qualitative Interviews: Process Evaluation

One-on-one semi-structured interviews were conducted in person or via telephone. Interview questions explored the acceptability and satisfaction with the ABW designed workplace, and its impact on workplace movement, eating behaviours, productivity, and workplace satisfaction. 

### 2.7. Statistical Analyses 

Data were processed and analysed using the Statistical Package for the Social Sciences (SPSS; version 23; IBM Corp, 2012, Armonk, New York, NY, USA) and STATA (version 15; StataCorp LP, College Station, TX, USA). Descriptive statistics were used to describe the socio-demographic characteristics of the sample (see Table 1), and ABW participants’ perceptions of changes to movement and their reported use of height-adjustable desks. Statistical significance was set at *p* < 0.05 for all analyses.

Differences in baseline age, sex, and outcome behaviours between participants with complete accelerometer data and those with only baseline accelerometer data were examined using independent *t*-tests and chi-square tests. Differences in raw means between the ABW and comparison participants at baseline and follow-up were calculated using *t*-tests (or Welch *t*-test) and chi-square tests. Differences between baseline and follow-up within groups were examined via paired *t*-tests. Linear mixed models with restricted maximum likelihood estimates were used to examine the intervention effects (group by time interaction) as these models are able to manage missing data [29] and therefore maximize the small sample size. The interaction between workplace and time provided an estimate of the relative change in the outcome at the ABW workplace compared to the comparison workplace between baseline and follow-up. The mixed models controlled for the potential confounders of age and sex. The analytic sample therefore included participants who had full data for covariates and the specific outcome of interest (either baseline or follow-up). All outcomes were tested for normality. SED minutes were log transformed and then standardised to obtain normality at baseline and follow-up. Effect sizes were calculated using Cohen’s *d* on change scores from the raw data of each variable of interest with values around 0.20, 0.5, and ≥0.80 representing small, moderate, and large effect sizes, respectively [30]. 

Thematic analyses were used to summarize the qualitative data [31] as outlined by Braun and Clarke [31]. The interview transcripts were initially read (Phase 1) and hand coded by two researchers (434 codes were assigned and there was an overall inter-rater reliability of 97% between the two researchers) (Phase 2). The data were then grouped into sub-categories using NVivo qualitative data software version 11 (e.g., snacking, stair use; Phase 3). Sub-themes were created based upon the sub-categories (e.g., healthier eating, increased movement; Phase 4) which allowed for the classification of key themes [31]. 

## 3. Results

Baseline data were collected from 146 participants (56% ABW participants; Figure 1) who were on average 40.1 (±8.5) years old. The majority were female (72%, worked full-time, were in the healthy weight range, and had a university/postgraduate level of education (see Table 1). The only significant difference in demographic characteristics between ABW and comparison workplace participants was a higher percentage of males in the ABW sample. 

Overall, 112 participants provided data at follow-up (54% ABW participants). Reasons for this attrition included leaving the workplace, on leave, sick/injured, or no reason provided (Figure 1). There were no significant differences in sex, age, or baseline time spent sedentary, in light-, or moderate-to vigorous-intensity physical activity between participants that had valid accelerometer at baseline and follow-up and those who only provided baseline data.

### 3.1. Workplace Activity

Time spent sedentary, in light- and moderate- to vigorous- intensity physical activity within the standardized eight-hour workday at baseline and follow-up is presented in Table 2. There were no significant intervention effects on any accelerometer-measured outcomes in linear mixed models; however, there were moderate effect sizes for reductions in sedentary time (Cohen’s *d* = −0.58) and increases in light-intensity physical activity (Cohens’ *d* = 0.55) and moderate-to-vigorous-intensity physical activity (Cohen’s *d* = 0.45). 

### 3.2. Organisational Support for Being Physically Active in the Workplace

There was significant intervention effect with higher levels of organisational support for being physically active in the workplace reported among the ABW workplace relative to the comparison workplace at follow-up compared to baseline. There were significant between-group differences in organisational support for being physically active in the workplace at both baseline and follow-up, with ABW participants reporting higher levels at both time points. Both the ABW and comparison participants reported significantly higher organisational support for being physically active in the workplace at follow-up compared to baseline.

### 3.3. Eating Behaviours

There was a significant intervention effects on the number of days participants had lunch with colleagues, with ABW participants reporting a higher frequency at follow-up compared to baseline, equating to nearly one extra day per week relative to the comparison group. Although no other significant within- or between-group effects were observed, there was a small effect size for an increase in the frequency of stopping for lunch (Cohens’ *d* = 0.23) after six to nine months of working in the ABW designed workplace. ABW participants significantly increased the frequency that they ate lunch with colleagues between baseline and follow-up. There were significant between group differences in the frequency of eating lunch with colleagues at baseline and follow-up with ABW participants reporting greater frequency at both time points. At follow-up, ABW participants also stopped for lunch significantly more often than participants from the comparison workplace. 

### 3.4. Productivity

Workplace productivity was higher among participants from the comparison workplace compared to the ABW workplace at baseline and follow-up. Between baseline and follow-up, workplace productivity significantly increased among participants from the comparison workplace but remained the same for the ABW group, resulting in a non-significant intervention effect, but a small negative effect size (Cohens’ *d* = −0.28).

### 3.5. Workplace Satisfaction 

There was a significant intervention effect on satisfaction with the physical environment with greater increase in satisfaction among the ABW group, relative to the comparison group at follow-up, which equated to a large effect size (Cohens’ *d* = 0.84). No other significant within- or between-group effects were observed for overall job satisfaction, relationship with co-workers, or relationship with supervisors. However, there were small-to-moderate effect sizes for improved satisfaction with the overall job (Cohens’ *d* = 0.38) and improved satisfaction with relationships with co-workers (Cohens’ *d* = 0.41).

### 3.6. Perceived Changes in Workplace Sitting and Use of Height-Adjustable Desks 

At follow-up, approximately half (49%) of the ABW participants reported that they had decreased their sitting while at work, and the majority perceived that they had increased the time they spent standing (62%) and walking/moving (74%), as well as the number of breaks in sitting they took (67%) during working hours compared to one year prior. At follow-up, 30% of ABW participants (*n* = 16) reported that they never used the height-adjustable desks, 34% used them less than once/week (*n* = 18), 28% one or two days/week (*n* = 15), and 8% three or more days/week (*n* = 4). For participants who reported using the desks at least once/week, 46% reported standing for most of the time, 22% half of the time, 11% for a quarter of the time, 11% regularly changed between sitting and standing, and 11% did not report standing at all. The majority of ABW participants (68%) reported wanting to continue to use the height-adjustable desks more often. Almost two-thirds (61%) reported that unavailability was the main barrier to using the height-adjustable desks.

### 3.7. Qualitative Study

**Figure 2:**
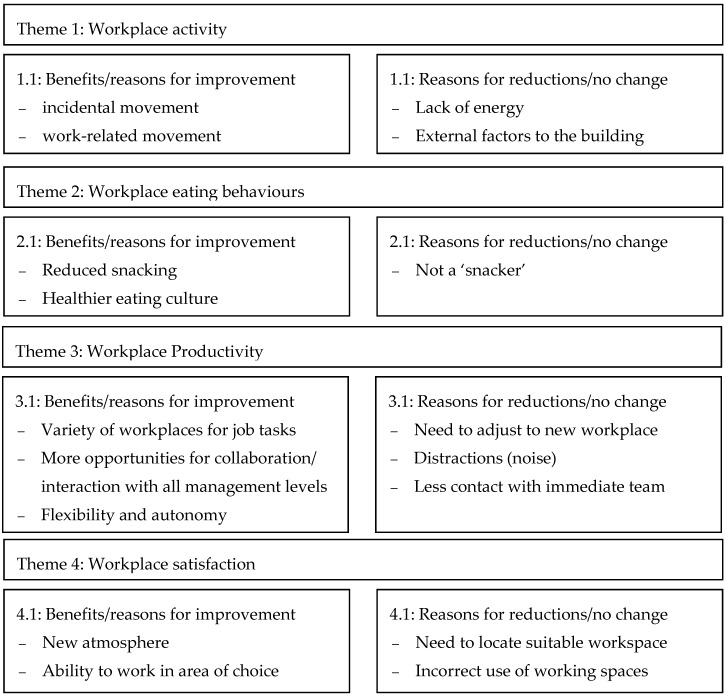
Themes and sub-themes constructed from the qualitative data.

Four key themes emerged from the qualitative data analysis including the impact or changes to workplace activity, workplace eating, productivity, and satisfaction with the workplace environment after moving to the ABW designed workplace (Figure 2). Data saturation was achieved prior to the final interview.

#### 3.7.1. Theme 1: Perceived Changes to Workplace Activity 

The majority (i.e., more than half) of ABW participants interviewed believed they were more active at work because the ABW environment was more conducive to movement. Movement was often described as incidental and in response to a work-related goal (e.g., finding a colleague, moving to a different place to work) or eating.

“Certainly [I] do more moving around, to different spaces over the course of the day, moving up and down the stairs… moving around is quite easy”.

(Male, 45 years)

Only a few of the interviewed participants believed that their movement and/or sitting habits had decreased or had not changed, with some attributing this to a lack of energy or factors external to the ABW building (e.g., no nearby shops to walk to for coffee, no walking paths).

“At the moment, we are in the middle of a paddock...so my activity levels at lunch time are lower.”

(Female, 30 years)

#### 3.7.2. Theme 2: Perceived Change to Workplace Eating Behaviours 

Most (i.e., more than half) interviewed participants associated the ABW environment with favourable impacts on eating behaviours, particularly reduced snacking and junk food consumption because of the policy banning eating at desks.

“I used to snack whenever, but the fact that I’m forced to have breaks I take them now. I’m probably eating less chocolate, because I am having the morning and lunch breaks at proper times, instead of whenever.”

(Female, 31 years)

Participants also believed there was a healthier eating culture in the ABW building, which was attributed to healthy eating alternatives in the ABW building (e.g., no vending machines, having sandwich options) and a peer influence due to eating together in common hub areas.

“We can’t just sit at our desks and hide. Other people see what you eat, its peer pressure in a way, but it’s good peer pressure. Everyone is sort of encouraging each other to be healthy.”

(Female, 25 years)

Those who did not view themselves to ‘snack’ did not consider their eating patterns to have changed. One participant described a reduction in their fruit consumption, as they could not eat at their desk.

#### 3.7.3. Theme 3: Perceived Changes to Workplace Productivity

Many interviewed participants believed that the ABW environment had productivity benefits. This was commonly attributed to the variety of workspaces available to meet different working needs, such as quiet focus areas and areas designed for collaboration.

“Productivity wise [ABW] has been fantastic…having plenty of spaces to meet is a big help.”

(Female, 23 years)

It was also recognised that working in the same building, using different desks, having formal and informal workspaces available, and eating in common hub areas allowed for greater opportunities to collaborate. Employees felt this enabled them to get to know, directly interact, and work with colleagues, particularly those outside immediate teams, and across departments. Some participants believed the greater opportunity to collaborate enhanced communication, sharing of knowledge, and task efficiency.

“There is a lot more people interacting, people who wouldn’t normally interact. [The] sharing of knowledge and relationships are enhanced. When you need to work with someone, you know them better and can work quicker.”

(Female, 50 years)

Participants noted that they enjoyed these greater interactions from both a social and work perspective, and particularly from different levels of management. They discussed that this made them feel valued and that their workplace felt friendlier.

“Because we are all in the same building together we are all equal. The managers, no one has different desks or offices. We are interacting more with mangers and they are waving hello and stuff like that. You feel valued on that perspective.”

(Female, 25 years)

The greater flexibility and autonomy to structure the work day to suit individual needs was also believed to enhance productivity.

“It’s more relaxing. It’s flexible here, [you] feel productive, [and] can structure your time and day on how it suits you best.”

(Female, 23 years)

A small number of participants felt that the ABW environment negatively influenced productivity. Negative impacts cited included needing time to adjust to the new ways of working, distractions in the open-plan office (e.g., noise), and lost time finding desks and colleagues. Some participants acknowledged that while the organization was more connected, they felt less connected with their immediate team members, which they believed adversely impacted team morale and productivity.

“I really miss our team. We have the same language and so we can talk and share and help each other out. I miss the informal catch-ups.”

(Female, 41 years)

#### 3.7.4. Theme 4: Satisfaction with Workplace Environment 

Most interviewed participants reported liking the new ABW working environment, atmosphere and way of working.

“I do like coming into the new building. It has invigorated things.”

(Female, 30 years)

Aspects of the workplace that participants particularly liked included the ability to seek out a quiet ‘Focus’ space to concentrate and the diversity of working spaces and the ability to choose spaces based on how they wanted to work.

“It is nice to work in different spaces and with different people.”

(Female, 23 years)

Participants who voiced dissatisfaction with the environment mostly associated this with trying to find a desk and frustrations with the incorrect use of working spaces (e.g., people talking in the focus rooms, sitting at standing desks). A small proportion were unhappy about not having their own personal working space.

“I think the Focus here isn’t used correctly yet. The experience I’ve had, I’m in a Focus booth, and someone else is next door, but they will have someone else come and have a meeting. Which is not how the space is meant to [be] used.”

(Male, 54 years)

## 4. Discussion 

This is one of few studies to evaluate the impact of structural and policy workplace changes brought about by ABW on workplace movement and productivity, and only the second to include a comparison workplace. It is also the first study to our knowledge to examine eating behaviours, support for being physically active in the workplace and workplace satisfaction after moving to an ABW designed workplace design. While there were no significant changes in the sedentary time and physical activity in the workplace, the moderate effect sizes suggest that an ABW designed workplace may improve the amount of time employees spend moving around in their workplace. Improvements were also seen in ABW employees’ eating behaviours, organisational support for being physically active in the workplace, satisfaction with their job, the physical work environment and their relationship with colleagues, while there was a small decline in productivity.

Although statistically non-significant, ABW participants slightly reduced their sedentary time at work per eight-hour workday (by 4.4 min) after six to nine months compared to the comparison group who increased their workday sedentary time (by 8 min). Furthermore, the ABW participants increased their light and moderate intensity physical activity slightly (by 2.6 and 1.8 min per workday respectively), while the comparison group performed less activity after six to nine months (6.3 and 1.7 min less per workday respectively). These findings are in line with studies of Australian [20] and Swedish [21] employees. They also suggest that improvements in workplace behaviours occur after a longer period of time working in an ABW designed office, when any novelty of the change may have worn off and employees have settled into the new working style. Participants from the ABW workplace in the current study also self-reported improvements in workplace behaviours. These changes, combined with the objective measures, are not surprising given participants also reported a significant improvement in organisational support for workplace movement, potentially making standing and moving more acceptable. Importantly, the current study used hip-worn accelerometers, which have a limited ability to differentiate between low-intensity behaviours [32] and postural changes (e.g., sitting vs. standing). Thus, time spent standing still (including time standing at a sit-stand desk) may have been captured within the sedentary time estimates, thus underestimating potential changes. It will be important to capture such postural shifts in further research.

The policy prohibiting eating at the desk was a key policy change associated with ABW. This is the first study to examine the influence of ABW on eating behaviours. Findings showed that ABW employees ate lunch more often with colleagues (about one day a week more), relative to the comparison workplace. This may have contributed to benefits identified in our qualitative findings such as increased social interaction and therefore improved relationships with colleagues. It may also have encouraged incidental activity, particularly amongst employees who traditionally ate at their desk. Qualitative data also suggested that dietary improvements may have been due to participants’ reductions in junk food snacking and a healthy workplace culture around eating. The results highlight the potential for workplace eating policies to not only promote better nutrition but also adds to the additional evidence called for by a recent review of environmental interventions targeting workplace eating behaviours [33]. Future research examining the impact of ABW on the intake of healthy and junk foods, and possible implications for movement and social benefits, over longer follow-up periods is warranted.

Following the implementation of ABW, perceived work productivity was lower relative to the comparison workplace. This is in contrast with previous findings examining self-reported productivity after a four-week trial of an ABW designed workplace [20] but in line with studies examining open-plan or shared offices [34] and suggests a need to address unique barriers to productivity in the new environment. While it is possible that ABW employees may require a longer time to adjust to the new environment and ways of working, and improvements may be seen after longer periods, several barriers were identified. ABW participants in the current study felt frustrated due to lost time finding desks and colleagues, noise in the workplace, and a period of time needed to adjust to the new ways of working, all of which may have adversely influenced productivity. The use of objective measures of productivity (e.g., organisational outputs) is also needed to strengthen this evidence. There was a moderate positive effect on ABW participants’ satisfaction with their co-workers yet the qualitative findings suggest that many believe intra-team-related work declined in the new ABW environment. Some ABW employees suggested that although they were interacting with more people than they used to, they felt less connected with the people in their own team, which may have also influenced work productivity. These findings align with Kim et al. [35], who found that workplace productivity was lower when there was limited interaction with colleagues, inability to adjust and personalise ones’ workspace and limited storage space, all of which occurred within the ABW designed workplace. It may therefore be important for employers considering a transition to the ABW design and principles to consider how to enhance team-specific connections and potentially encourage some personalisation of employees’ workspace. 

The ABW employees reported higher levels of satisfaction with the physical work environment relative to those in the comparison workplace. ABW employees liked the new environment, particularly the diversity and flexibility in ways of working, and this may have contributed to the improvement in overall job satisfaction. Few negative reactions to the physical workplace emerged from the qualitative data and these typically related to not having personal workspaces, difficulty finding suitable desks, and the improper use of working. Employers planning to move to an ABW design may benefit from including additional prompts to encourage staff to use the appropriate workspace (e.g., point-of-choice prompting software [36]) or workshops on work space etiquette and intervention adoption [37].

This is the first study to evaluate the impact of ABW on a combination of workplace movement, eating behaviours, and workplace outcomes. There are, however, a number of limitations that should be considered. Firstly, this study was conducted over a six to nine month follow-up period and it is possible that additional time is needed to adjust to the ABW design and working principles. Additional follow-up data-collection time points are therefore required to measure possible changes over longer periods of time. In addition, although the sample was larger than any previous studies examining the impact of ABW designs [20] and had a strong retention rate (78%), the sample size was small, comprised only one workplace per group, and there were poor participation rates, particularly amongst the comparison group. As such, participants may not be representative of the broader work team. Studies with larger sample sizes and more workplaces are needed. There was also poor compliance with the accelerometer and logbook protocols, particularly at follow-up, with a substantial proportion of work hours requiring imputation. However, there were no significant differences between those with complete versus incomplete data at both time points. As noted above, the monitor used does not adequately distinguish between sitting and standing (two common postures in the work environment), and the five-day protocol may not have been sufficient to capture habitual behaviour. A further limitation was the introduction of some ABW policies prior to the relocation to the new workplace. This may have diminished our ability to show true effects of ABW on the outcomes explored, as suggested by the higher baseline rates of positive behaviours observed in the ABW group. The strengths of this study include the objective measure of workplace physical activity and sedentary behaviour; and importantly, the inclusion of a comparison group enabling both within and between-group differences to be examined. The addition of qualitative data also provides valuable process evaluation information that could be used to assist employers and employees when moving to an ABW designed workplace. Future research examining the impact of ABW should include objective measures of productivity and employee wellbeing (e.g., absenteeism) and objective measures of posture to build upon these findings. We note that we attempted to collect inclinometer data in the current study; however, compliance was poor and therefore data are not reported. Future studies should also consider strategies to increase monitor compliance.

## 5. Conclusions 

In conclusion, findings suggest that transitioning to an ABW-designed workplace may improve workplace sedentary time and physical activity, eating behaviours, support for being physically active in the workplace, and workplace satisfaction. Future research with larger samples, over longer follow-up periods, and with objective measures of sitting and standing is needed. Further exploration of other environmental (i.e., more height-adjustable desks), organisational, social, and individual-level strategies that may compliment the effective use of ABW and promote employee health and workplace outcomes is also warranted. 

## Figures and Tables

**Table 1:** Participant demographics at baseline

Demographic	ABW Workplace *n* = 79	Comparison Workplace *n* = 61
Age (years) ^a^	39.1 (10.2)	41.4 (9.9)
Sex (male) ^b^	35.9%	16.4% *
Work capacity ^b^		
Full-time	84.8% (67)	72.1% (44)
Part-time	15.2% (13)	27.9% (17)
Weight status ^b^		
Healthy weight	46.75 (36)	55.93 (33)
Overweight/obese	53.25 (41)	44.07 (26)
Highest level of education ^b^		
≤ Year 12	5 (4)	12 (7)
Certificate/diploma/apprenticeship	26 (20)	38 (23)
University/postgraduate	69 (54)	51 (31)

Notes: ^a^ Mean (SD); ^b^ % (*n*); * significant difference (*p* < 0.05) as determined by *t*-test or chi-squared test.

**Table 2:** Mixed models (coefficients [*b*] 95% CI, *p*-value) examining the impact of ABW on workplace activity, eating patterns, productivity, and workplace satisfaction outcomes.

	ABW Workplace		Comparison Workplace		Intervention Effect ^b^ (Workplace × Time)		Effect Size (Cohen’s *d*)
	PreMean (SD)	PostMean (SD)	PreMean (SD)	PostMean (SD)	*b* (95%CI)	*p*-Value	
Objective measures	*n* = 79	*n* = 62	*n* = 51	*n* = 45			
Workplace activity (min/8 h workday)							
Sedentary time ^a^	387.5 (27.5)	383.1 (41.6)	379.8 (29.5)	387.8 (29.6)	−0.4 [−1.0, 0.3]	0.23	−0.58
Light intensity PA ^a^	74.3 (22.5)	76.9 (37.4)	77.9 (24.7)	71.7 (25.2)	10.9 [−5.6, 27.4]	0.20	0.55
Moderate-to-vigorous-intensity PA ^a^	**18.2 (8.4)** *	19.9 (9.7)	**22.2 (10.8)** *	20.6 (10.9)	3.2 [−2.5, 8.9]	0.28	0.45
Survey measures	*n* = 79	*n* = 61	*n* = 53	*n* = 45			
Organisational support for PA in the workplace							
Organisational support (support/past month)	**32.5 (5.2)** *^,¥^	**35.5 (4.0)** *^,¥^	**30.2 (4.6)** *^,¥^	**31.7 (4.5)** *^,¥^	**2.0 [0.3, 6.7]**	**0.03**	0.50
Eating behaviours							
Stopped for lunch (days/work week)	3.6 (1.5)	**4.1 (1.5)** *	3.5 (1.5)	**3.5 (1.6)** *	0.4 [−0.3, 1.1]	0.27	0.23
Lunch with colleagues (days/work-week)	**1.9 (1.6)** *^,¥^	**2.6 (1.4)** *^,¥^	**1.0 (1.2)** *	**0.8 (0.8)** *	**0.8 [0.2, 1.4]**	**<0.01**	0.52
Snacking between meals (days/work-week)	3.0 (1.8)	2.4 (1.9)	3.1 (1.7)	3.1 (1.9)	−0.4 [−1.2, 0.4]	0.32	−0.16
Productivity							
Productivity score (satisfaction/past work-week)	7.7 (1.0)	**7.7 (0.9)** *	7.9 (0.9) ^¥^	**8.1 (0.8)** *^,¥^	−0.3 (−0.6, 1.1)	0.18	−0.28
Satisfaction ratings							
Overall job	**7.0 (1.8)** ^¥^	7.2 (1.4) ^¥^	7.6 (1.9)	7.2 (1.7)	0.6 [−0.2, 1.4]	0.15	0.38
Relationship with co-workers	7.7 (1.6)	7.9 (1.7)	8.1 (1.6)	7.9 (1.6)	0.5 [−0.2, 1.2]	0.18	0.41
Relationship with supervisors	7.7 (1.8)	7.8 (1.9)	7.9 (1.8)	8.04 (1.7)	0.1 [−0.6, 0.8]	0.83	0.13
Physical environment	**5.0 (2.5)** ^¥^	**7.7 (2.4)** *^,¥^	5.4 (2.1)	**5.9 (2.3)** *	**2.1 (1.0, 3.3)**	**<0.01**	0.84

^a^ Mean times and frequencies are data standardised for 8-h worktime; ^b^ Results from linear-mixed models: all analyses examined workplace by time interaction and adjusted for age and sex. * indicates significant between group differences at baseline or follow-up (*p* < 0.05) using *t*-tests (or Welch *t*-test) and chi-square tests in **bold**; ^¥^ indicates within group difference between baseline and follow-up (*p* < 0.05) using paired-*t*-test in **bold**. Linear mixed model performed using transformed data. PA = physical activity.
